# Neural correlates of word learning in children

**DOI:** 10.1016/j.dcn.2019.100649

**Published:** 2019-04-28

**Authors:** Atsuko Takashima, Iske Bakker-Marshall, Janet G. van Hell, James M. McQueen, Gabriele Janzen

**Affiliations:** a Radboud University, Donders Institute for Brain, Cognition and Behaviour, P.O. Box 9101, 6500 HB, Nijmegen, the Netherlands; b Radboud University, Behavioural Science Institute, P.O. Box 9104, 6500 HE, Nijmegen, the Netherlands; c Max Planck Institute for Psycholinguistics, P.O. Box 310, 6500 AH, Nijmegen, the Netherlands; d Pennsylvania State University, Department of Psychology, University Park, PA, 16802, USA; e University of Oxford, Department of Clinical Neurosciences, Wellcome Centre for Integrative Neuroimaging, FMRIB, John Radcliffe Hospital, Headley Way, Oxford, OX3 9DU, United Kingdom

**Keywords:** AAL, automated anatomical labeling, CLS, Complementary Learning Systems, fMRI, functional magnetic resonance imaging, GLM, general linear model, ICs, independent components, IFG, inferior frontal gyrus, IPL, inferior parietal lobe, L1, first language, L2, second language, MNI, Montreal Neurological Institute, pMTG, posterior middle temporal gyrus, ROI, region of interest, STG, superior temporal gyrus, SVC, small volume corrected, Novel word memory, Brain development, Hippocampus, Lexicalization, Consolidation, fMRI

## Abstract

Memory representations of words are thought to undergo changes with consolidation: Episodic memories of novel words are transformed into lexical representations that interact with other words in the mental dictionary. Behavioral studies have shown that this lexical integration process is enhanced when there is more time for consolidation. Neuroimaging studies have further revealed that novel word representations are initially represented in a hippocampally-centered system, whereas left posterior middle temporal cortex activation increases with lexicalization. In this study, we measured behavioral and brain responses to newly-learned words in children. Two groups of Dutch children, aged between 8–10 and 14–16 years, were trained on 30 novel Japanese words depicting novel concepts. Children were tested on word-forms, word-meanings, and the novel words’ influence on existing word processing immediately after training, and again after a week. In line with the adult findings, hippocampal involvement decreased with time. Lexical integration, however, was not observed immediately or after a week, neither behaviorally nor neurally. It appears that time alone is not always sufficient for lexical integration to occur. We suggest that other factors (e.g., the novelty of the concepts and familiarity with the language the words are derived from) might also influence the integration process.

## Highlights

• We measured brain activity patterns for recognizing newly trained words in children.
• Retrieval related activation in the hippocampus decreased a week after training.
• Lexical integration effect was not observed even after a delay of 1 week.
• Younger group used right hemisphere more whereas teens used left hemisphere more.

## 1 Introduction

We encounter novel words throughout our lives and our vocabulary continues to grow at least until we are 60 years old (Hartshorne and Germine, 2015). Although vocabulary size and growth differ across ages and educational levels, children increase their vocabulary size throughout their school years. How do they retain and integrate these novel words in their mental dictionary? Do children differ from adults in the way they learn novel words? How do their brains store novel word memories? To date, these key questions have remained largely unanswered, and especially the way in which novel words are learned and remembered at the neural level is not well understood. This study investigates the neural correlates of novel word learning in school-aged children.

Acquisition of novel linguistic information can rely on multiple memory systems as proposed in the declarative/procedural model (Ullman, 2016). Vocabulary learning more likely utilizes the declarative system due to its explicit instruction in the associations between word forms and their meanings, but grammatical learning can also depend on the declarative system, especially for non-native languages (Ullman and Lovelett, 2018). One line of research on word learning in young adults has focused on how the mental representation of words changes as a consequence of time and consolidation (Davis and Gaskell, 2009; Gaskell and Dumay, 2003). The Complementary Learning Systems account (CLS) of word learning put forward by Davis and Gaskell (Davis and Gaskell, 2009) is based on the CLS account proposed in the memory literature (McClelland et al., 1995) where novel words are initially sparsely encoded, separately from existing lexical knowledge, and gradually integrated into a pool of other memory representations over time. In this process, the word representation is not only stabilized such that the word can be retrieved later in time (consolidation), but links between the novel word and the associated words in the mental lexicon are formed (integration/lexicalization).

In line with this theory, Gaskell and colleagues have identified two steps in how we respond to novel words. Immediately after learning a set of new words, we are able to retrieve their episodic memory traces, which allows us to recognize the studied words in a test. A subsequent period of consolidation, however, is necessary for these novel words to become integrated into the mental lexicon. It is this integration process that is hypothesized to underlie the emergence of interaction effects between novel and existing words, for instance, interference between phonological neighbors or facilitation of processing of semantically related words (Bakker et al., 2014; Dumay and Gaskell, 2007, 2012; Gaskell and Dumay, 2003; Tamminen and Gaskell, 2008, 2013; van der Ven et al., 2015). This is similar to the distinction between lexical configuration and engagement made in other models of word learning (Leach and Samuel, 2007; Storkel and Lee, 2011). These models state that it is only after the novel word has achieved lexical engagement that it begins to interact with other words in the mental lexicon, which can extend to words in a second language (L2). Although some studies have found no integration effect and concluded that the L2 lexicon is separate from the first language lexicon (L1) (e.g., Qiao and Forster, 2017), other models do consider an integrated lexical system (discussed e.g. in Sabourin et al., 2013), and many bilingual studies have shown cross-linguistic priming effects (reviewed in van Hell and Kroll, 2012; Wen and van Heuven, 2017), providing evidence that L1 and L2 words do interact with each other.

Following up on these behavioral findings, neuroimaging studies have found that brain signatures of novel words also change as a function of time (Bakker-Marshall et al., 2018; Bakker et al., 2015a, 2015b; Takashima et al., 2014, 2017; Tamminen et al., 2010). Animal models, human patient case studies, as well as functional neuroimaging studies of memory consolidation have revealed that memories are represented in different structures of the brain before and after consolidation (Alvarez and Squire, 1994; Squire, 1986; Squire et al., 2015). In line with this systems-level memory consolidation theory, studies of word learning have identified that the medial temporal lobe, and especially the hippocampus, is more engaged in the initial stage of novel word learning (Breitenstein et al., 2005; Davis et al., 2009), but that with time and consolidation, cortical structures become more engaged (Bakker-Marshall et al., 2018; Takashima et al., 2014, 2017). In particular, we have shown that the left middle temporal lobe, often discussed as a memory area for word representations (Gow, 2012; Hagoort, 2013; Hickok and Poeppel, 2004, 2007), showed an increase in engagement at delayed test (Bakker-Marshall et al., 2018; Takashima et al., 2017), and the level of engagement correlated with behavioral lexicalization effects (Bakker-Marshall et al., 2018; Takashima et al., 2014).

Research on school-aged children has shown that, after a period of sleep, children also show a lexicalization effect with novel words. Better memory performance was observed after a delay period including sleep (Brown et al., 2012). Lexicalization effects in children were observed after a night of sleep, similar to those found in the young adult population (Henderson et al., 2015, 2012, 2013a; Henderson et al., 2013b; van der Ven et al., 2017). Furthermore, specific sleep processes, such as slow oscillations and spindles, which have been found to be associated with memory consolidation in adults, are also observed in children (Smith et al., 2018; Wilhelm et al., 2013).

However, studies on the neural correlates of this consolidation process with novel words in children are scarce (Landi et al., 2018, tested adolescents). Despite the support for the CLS framework in developmental populations, it is currently not known whether the behavioral effects are underpinned by the same neural mechanisms at different ages (James et al., 2017). Given the similar findings in behavioral and sleep studies between young adults and school-aged children, it might appear likely that the same consolidation process is taking place across ages. But because children's brains are still in a state of protracted development that extends to puberty and beyond, and because different structures of the brain have different maturation profiles (Ghetti and Bunge, 2012; Menon et al., 2005; Paus, 2005; Schmithorst et al., 2007), we might observe a response to novel words that is different from the pattern observed in the young adult population. Especially since there is a protracted development of the hippocampus in middle childhood (ages 6–11 years) (Ghetti and Bunge, 2012), we expected younger children to show a weaker consolidation/integration effect compared to teenagers.

In order to investigate the neural responses to newly learned words in children, and how these might change as a function of time, we trained two groups of school-aged children before and after puberty on 30 novel words with novel concepts. We then measured their brain responses with functional magnetic resonance imaging (fMRI), using a lexical decision task to tap into word-form memory representations, once directly after training and again after a week’s delay. We further measured their behavioral responses (in the lexical decision task in the scanner and in free and cued recall tasks outside of the scanner) to test memory for both the forms and the meanings of the new words. Outside of the scanner, we also tested for a lexicalization (integration) effect using a semantic priming task, in which the trained novel words were used as primes to native-language target words that were either semantically related or unrelated to the primes. We assumed that facilitation of target-word processing through activation of the prime’s semantic information would only appear once the novel words were incorporated into the mental lexicon. Based on a prior study in school-aged children (van der Ven et al., 2017), we expected that the priming effect would be observed only after a delay including sleep. As for the brain responses, we expected more involvement of the hippocampus in the first scanning session than in the second one. In contrast, we expected increased left posterior middle temporal lobe activation in the second session relative to the first one.

## 2 Materials and methods

### 2.1 Participants

Forty-eight typically developing and native Dutch-speaking children participated in the experiment. Half of the children were aged between 8 and 10 years (Young group; *M* = 9 years 9 months at the time of screening), and the other half were aged between 14 and 16 years (Teen group; *M* = 15 years 6 months at the time of screening; see Supplementary Table 1 for details). The age range for children in the Young group was chosen such that the children were as young as possible while still having the motivation to follow the protocol of the study, a good enough attention span and the willingness to lie still in the scanner while doing the task. The age range for the Teen group was chosen because at this age the hippocampus has matured to a similar state to that of young adults (Ghetti and Bunge, 2012) but the prefrontal cortex is still largely under development, and thus may show a differential pattern to that found in prior studies targeting the young adult population. All participants were screened prior to the actual experiment to check for all inclusion and exclusion criteria. They were all right-handed, had normal or corrected-to-normal vision and normal hearing, and reported no dyslexia or other language impairments or a history of neurological impairments. All children had no or very little knowledge of the Japanese language or Japanese culture. Furthermore, all had no contra-indications to magnetic resonance imaging (MRI). Both children and parents were informed about the study, and gave written consent and were compensated for their participation. The study was approved by the ethical committee of the faculty of social sciences, Radboud University (ECSW2013-0410-134, CMO waiver 45516.091.13).

### 2.2 Stimulus materials

#### 2.2.1 Japanese words

Thirty Japanese nouns depicting objects were selected (e.g. *tanuki*, “A small badger-like animal that appears in Japanese fables. They like to trick people”; see Supplementary Table 2 for the complete list of stimuli). All objects were unfamiliar in Dutch culture as established in a pilot study (adults *n* = 10, children *n* = 3). The selected words were between two to four syllables (*M* = 2.6). For each word, two pictures depicting its referent were obtained from internet sources, and two versions of Dutch definitions were created: a long version (range 6–21 words, *M* = 13.4) and a short version (range 2–6 words, *M* = 3.4). Audio files of each word, spoken by a Dutch female speaker, were created using PRAAT (Boersma and Weenink, 2011). By providing both the image and the verbal definition for each novel word, we aimed to clarify the concept of the novel word, compensating for potential ambiguities in one format by the other.

#### 2.2.2 Dutch words

For the lexical decision task in the fMRI scanner, 30 common Dutch nouns were used (number of letters *M* = 6.5, number of phonemes *M* = 6.0, frequency per million *M* = 10.1, range 1–49). Audio files of these words were produced by the same Dutch female speaker as the Japanese words. For the priming test, two semantically associated Dutch words were selected for each Japanese word. The selected target words could be categorically or functionally associated with the trained novel words, and they were words known to 8-year-olds. For example, for the Japanese word *geta* which means “a high soled wooden sandal”, the Dutch words *slipper* (slipper) and *schoen* (shoe) were selected. These words did not appear in the definition of the associated Japanese words. The two selected words were assigned to either list 1 or 2 and one list was used at immediate test and the other at delayed test. The assignment of the list to the session was counter-balanced across participants.

#### 2.2.3 Pseudowords (Japanese/Dutch)

For stimuli to serve as pseudo-Japanese words during the lexical decision task in the scanner, 30 non-studied Japanese words were used (range 2–4 syllables, *M* = 2.9). Additionally, 30 Dutch pseudowords were created from each word used for the lexical decision task in the scanner (mentioned in the section 2.2.2 Dutch words) by substituting two to five letters (number of substituted letters *M* = 3.0, e.g. *fapero* for camera). Furthermore, for every Dutch word used in the priming test, a pseudoword was created by substituting 1–5 letters (*M* = 2.2, e.g. *skepper* for slipper). All created pseudo Dutch words followed the phonotactic rules in Dutch. Half of the pseudowords prepared for the priming task were assigned to list 1 and the other half to list 2. Pseudowords used in the scanner were produced by the same speaker who pronounced the Japanese and the Dutch words.

### 2.3 Procedure

**Fig. 1:**
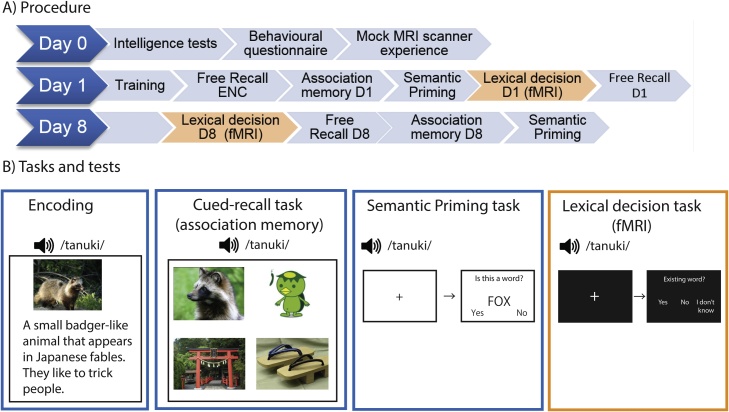
*Experimental procedures and tasks*. A) Experimental procedures: The experiment consisted of 3 sessions. Day0: Intake and practice in the mock scanner. Day1: Training and testing of the novel words. Day8 (one week after Day1): Testing of the trained novel words. ENC = Encoding; D1 = Day1; D8 = Day8. B) Example screens during the training and test. During Encoding, the children heard the word and its definition through the speaker while the picture and the definition was also shown on the screen. During the cued-recall task, children heard the trained word, and had to choose the corresponding picture between the 4 options on the screen. The example on the screen shows a trial from the Similar condition at test. During the Semantic priming task, the children heard a word and then saw a word on the screen. They were instructed to indicate by button press whether the word on the screen was existing or not. During the Lexical decision task in the scanner, the children heard the word, and they had to indicate whether the word was existing or not via a button press. See method section for details. Dutch words were used in the actual experiment, but for illustrative purpose the texts in the figures are in English.

The experiment took place over three sessions (Fig. 1A). On Day0 (screening), participants came to the lab to be checked for inclusion/exclusion criteria. Child participants were tested on their working memory span (forward and backward digit span, word list repetition), vocabulary size (Peabody Picture Naming Task: PPVT, Dutch version), and logical thinking (Raven’s Progressive Matrices). Their parents were asked to fill out a Child Behaviour Check List (CBCL). See Supplementary Table 1 for a summary of their demographic information. Following these tests, participants were familiarized with scanning procedures by placing them in a mock fMRI scanner with scanner sounds, and they completed a short task similar to that of the actual experimental task. No participant was excluded as a result of these procedures. Participants were informed about the outline of the actual training and test sessions and the two experimental sessions were planned.

On Day1 (experimental session1), participants were trained on 30 Japanese words and were subsequently tested both behaviorally and in the scanner. On Day8 (experimental session2), exactly one week after the training session, participants returned to the lab to be tested on the trained words. At the end of the session, participants were debriefed about the purpose of the study and the hypothesis being tested.

#### 2.3.1 Encoding and training of Japanese words

Training consisted of encoding the 30 Japanese words (spoken form of the novel word presented together with the corresponding image shown on the screen and definition written below the image and read out through the speaker) presented once each (Fig. 1B), followed by multiple training tasks, including cued recall (6 blocks: 3 blocks of four-alternative forced choice of a picture when cued with the word, and 3 blocks of three-alternative forced choice of a definition when cued with the word) with correct response feedback for each trial (correct choice marked as green with the spoken word repeated through the speaker), word repetition (upon hearing the word and seeing the image, the participants were instructed to repeat the word, once for each word), picture naming (a trained image appeared on the screen and the participant named the picture, correct word was presented via the speaker at the end of each trial), and free recall of the words. The training session ended with a second round of encoding where, for all trained words, the spoken word form together with the image and the definition was presented once each. See supplementary material 5 for details. Overall, through training, participants were exposed to each novel word a total of 16 times.

#### 2.3.2 Free recall test

To test their word-form memory, children were asked to recall as many trained Japanese words as possible and speak them out loud within a time limit of three minutes. Responses were recorded and scored offline. For each response, three points were given if the word was pronounced perfectly, two points if the word sounded very similar to the trained word (one or two phoneme difference), and one point was given for any word that sounded similar to one of the trained words (the scorer can guess the word to be one from the list). The sum of the weighted score ((number of trials coded as correct x 3) + (number of trials coded as very similar x 2) + (number of trials coded as similar x 1)) was used as the performance measure. We opted for this four-step scoring system because the phonotactics of the novel language were very unfamiliar to the participants, and we observed them having difficulty in pronouncing the words even when they seemed to know the word-forms. A third of the items were scored by both of the scorers to check for inter-rater reliability. Ambiguous pronunciations were discussed and scoring criteria were agreed upon before further scoring the rest of the items.

#### 2.3.3 Association memory test (Cued-recall test)

Children’s memory of the words’ meanings was tested. Upon hearing the trained word, participants were asked to choose the picture that fitted the meaning of the word from four picture options (Fig. 1B). All Japanese words from the training session were tested one at a time. Following the setup of the previous study (Takashima et al., 2017), half of the pictures were the same as in the training (Same condition). The other half depicted the same objects but were slightly different from the pictures used in the training (Similar condition). In this way, we tried to probe semantic memory rather than episodic sound-picture association memory. Participants were informed that some pictures were not exactly the same as the ones presented during the training session, but that they should choose the similar one if the meaning of the word matched the picture. No feedback was given. Reaction time (RT) was calculated as the time between the onset of the word and the button response. Assignment of the two picture lists to either the Same or Similar condition was counterbalanced across participants and days, such that the words presented in the Same condition on Day1 were presented in the Similar condition on Day8, and vice versa.

#### 2.3.4 Semantic priming (primed lexical decision)

To test if the novel words were integrated, a semantic priming task was administered. Children were presented with an auditory prime word followed by a visual target which was either a word or a pseudoword, and they were instructed to indicate whether the target item was a word or not (Fig. 1B). All prime words were trained Japanese words. Target words were divided into three conditions: related Dutch, unrelated Dutch, and pseudo-Dutch words. Related Dutch words were semantically related to the prime Japanese words (see section 2.2.2 Dutch word*s*, e.g. prime: */geta/*, target: SLIPPER, Supplementary Table 2). Unrelated pairs were created by recombining the prime-related target word pairs such that there was no longer a semantic relation between the prime and the target. Furthermore, two Dutch pseudowords were paired with each Japanese prime word. Pseudowords were derived from the target Dutch words (see section 2.2.3 Pseudowords). This resulted in 120 prime-target pairs, and these were divided into four blocks. The order of presentation of the pairs was semi-randomized such that half of the related target words appeared in the first half and the other in the second half of the task. This ensured that there were 30 trials of related pairs and 30 trials of unrelated pairs with different target words in the first half, and the same again for the second half. Prime words were not repeated within a block, and target words were not repeated within the same half (i.e., if a target word appeared in the first or the second block, the second appearance of that word was in either the third or the fourth block).

For every trial, a 500 ms blue fixation cross was followed by the auditory presentation of the prime word. The target word appeared on the screen 250 ms after the offset of the prime word. Children were instructed to indicate by button press whether the target was a word or a non-word, as accurately and as fast as possible. The Young group had 2000 ms to respond and the Teen group had 1000 ms to respond. The trial terminated with the response or when the response time limit was reached. The inter-trial interval was 1000 ms. After every block consisting of 30 trials each, children could take a short break, and were instructed to press a button when they were ready to proceed to the next block. Before the actual test, they were familiarized with the task using a practice set of prime-target words not used in the actual experiment.

#### 2.3.5 Lexical decision test (fMRI)

The lexical decision task took place inside the MRI scanner. First, the stimulus sound volume was adjusted so that the words were audible above the scanner noise. Then the actual experiment began. There were four conditions (Japanese trained words, Dutch words, Japanese pseudowords, and Dutch pseudowords) with 30 words in each condition, presented through in-ear headphones one word at a time in a random order. For every trial, after a jittered inter-trial interval of 2000–6000 ms, the fixation cross turned from white to blue indicating the upcoming presentation of the stimulus. The stimulus was presented auditorily 1000 ms after the fixation cross had turned blue, and children were instructed to indicate whether the stimulus was a word or not by pressing one of the three buttons – “Yes”, “No”, or “I don’t know”. There was no time limit for responding. Once a response was made, the trial proceeded to the next one. Trials with correct responses were categorized as correct for each condition. “I don’t know” responses were omitted from the behavioral data calculations. For fMRI data, incorrect and “I don’t know” responses were categorized as incorrect.

### 2.4 MRI data acquisition

FMRI data were recorded in a 3Tesla magnetic resonance scanner (Skyra, Siemens Healthcare, Erlangen, Germany) using a 32-channel head coil. For functional images, we used a T2*-weighted gradient multi-echo planar imaging sequence with the following parameters: repetition time (TR): 2.28 s, echo time: TE1 8.5 ms, TE2 19.6 ms, TE3 31 ms, TE4 42 ms, 36 slices, ascending slice order, 2.7 mm slice thickness, 0.3 mm slice gap, inplane matrix size: 64 × 64, inplane field of view (FOV): 192 × 192 mm, flip angle: 90°. Slices were angulated in an oblique axial manner to reach whole-brain coverage (except for a part of the parietal cortex and the cerebellum). Additionally, T1-weighted anatomical scans at 1 mm isotropic resolution were acquired with TR 2300 ms, TE 3.03 ms, flip angle 8°, and FOV 256 × 256 × 192 mm. A diffusion tensor imaging sequence was also recorded, but was not analyzed.

### 2.5 fMRI analyses

#### 2.5.1 Preprocessing

Functional images were first realigned to the first image, then multiple echo images were combined to one value per voxel for each data point (Poser et al., 2006). Echo combined volumes were then preprocessed using the functions in FSL (FMRIB Software Library, FSLv5.0, available at http://www.fmrib.ox.ac.uk/fsl). FSL-FEAT was used for slice timing correction, for the Brain Extraction Tool, and for spatial smoothing of 6 mm full-width at half maximum. Subsequently, independent components (ICs) were calculated using FSL-MELODIC, which further was fed into FSL-ICA-AROMA for detection of movement related independent components (ICs). Visual inspections of all ICs were performed by two investigators to mark artefact related ICs that were not detected by the ICA-AROMA procedure. All artefact related ICs were removed from the data using FSL-regfilt. Output from the above steps was further preprocessed using SPM12 (https://www.fil.ion.ucl.ac.uk/spm/) for coregistration of the functional images to the structural image, realigning and reslicing the functional images of the two sessions, segmentation of the structural image, and normalization of the functional images to Montreal Neurological Institute (MNI) space.

After careful manual inspection of the preprocessed data, five data sets from the young group were excluded from the analyses because they still contained obvious artifacts such as stripes in the brain volumes or volume displacement even after the realignment procedure. This left n = 19 for the Young group and n = 24 for the Teen group. No data was removed based on the participant’s behavioral performance measures.

#### 2.5.2 Subject level analysis

For each participant, a general linear model (GLM) was applied to the functional data of the two sessions. The time course of each condition was convolved with the canonical hemodynamic response function (duration set to 0 s) provided by SPM12, with the onset of each trial set at the beginning of the word. The regressors included conditions of interest (correctly responded trials for Japanese, Dutch, Pseudo-Japanese and Pseudo-Dutch, and all incorrect trials pooled, for each session) and 48 regressors of no interest for each session comprising of 6 motion regressors (translation and rotation in x, y, and z axis), 6 derivatives of the motion regressors, and 36 slice regressors, each corresponding to the mean slice intensity for each volume. For both Japanese and Dutch conditions, we created a contrast image < words – pseudowords > each, separately for the two sessions at the subject-level, and these contrast images were used in the group level analyses. To test for between-language differences across the two age groups, a contrast image < Japanese - Dutch > was created, and these contrast images were compared between the groups.

#### 2.5.3 Group level analysis

To test for effects of the within-subject factors Language (Japanese, Dutch) and Time (Day1, Day8), a flexible factorial design was used. A flexible factorial design allows one to compare main and interaction effects for the imaging data, taking into account the dependencies in the data that come from the same participant. We pooled data from both the Young and the Teen groups to test a general effect across age groups. To test for effects of the between-group factor, contrasts of interest were compared between the Young and the Teen groups using two-samples t-tests. The significance level was set at cluster-level *p* =  0.05 (family-wise error corrected for multiple comparisons), where the initial cluster defining voxel level threshold was set at *p* =  0.001, uncorrected. Since we were specifically interested in the role of the hippocampus and the left posterior middle temporal gyrus (pMTG) for retrieval of newly trained words, we further restricted our search volume to the bilateral hippocampi (anatomical area defined by the automated anatomical labeling (AAL) template (Tzourio-Mazoyer et al., 2002)) and the left pMTG (derived from a sphere of 20 mm centered at peak coordinate reported in our previous study on word consolidation (Takashima et al., 2017)) within the middle temporal gyrus as defined by the AAL template).

## 3 Results

### 3.1 Behavioral

#### 3.1.1 Free recall

Due to technical issues, data of two participants (one girl in the Young group, and one girl in the Teen group) were not recorded during the free recall test after the lexical decision task on Day1, and thus the statistics are based on n = 46 (Young n = 23, Teen n = 23). A repeated measures ANOVA with factors Group and Time on weighted score (max 90 points) revealed a main effect of Group (*F(1,44)* = 12.05, *p* =  .001, *η_p_^2^* = .22), with scores being higher for the Teen (*M*(*SE*) = 25.5(2.2)) than the Young group, (*M*(*SE*) = 15.0(2.2)). A main effect of Time (*F(1,44)* = 27.25, *p* <  .001, *η_p_^2^* = .38) showed that participants recalled more words on Day8 (*M*(*SE*) = 23.0 (1.7)) than on Day1 (*M*(*SE*) = 17.5(1.5)). There was no interaction effect (*p* =  .762).

#### 3.1.2 Association memory

##### 3.1.2.1 Accuracy

**Fig. 2:**
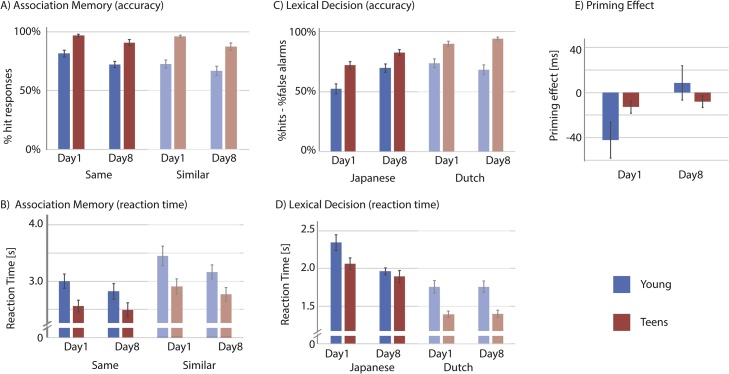
*Behavioral results*. Error bars denote standard error of the means.

Participants responded well above chance level (chance level = 25%) for both conditions on both days (Fig. 2A). A repeated measures ANOVA with Time (Day1, Day8) and Condition (Same, Similar) as within factors and Group (Young, Teen) as a between factor revealed main effects of Time (*F(1,46)* = 20.22, *p* <  .001, *η_p_^2^* = .31; Day1 *M*(*SE*) = 86.7 (1.4) %, Day8 *M*(*SE*) = 79.3 (2. 0)%), Condition (*F(1,46)* = 15.58, *p* <  .001, *η_p_^2^* = .25; Same *M*(*SE*) = 85.4(1.4)%, Similar *M*(*SE*) = 80.6(1.9)%), Group (*F(1,46)* = 40.37, *p* < .001, η_p_^2^ = .47; Young *M* = 73.2 (2.2) %, Teen *M*(*SE*) = 92.8 (2.2) %), as well as a 2-way interaction Condition × Group (*F(1,46)* = 4.70, *p* =  .035, *η_p_^2^* = .09). Post-hoc t-tests revealed that the Young group performed worse on the Similar condition relative to the Same condition when compared to the Teens (mean difference Same - Similar: Young *M*(*SE*) = 7.4 (2.4) % and Teen *M*(*SE*) = 2.2 (0.8) %; *t(28.2)* = 2.10, *p* =  .040), although this effect may be caused by the ceiling effect in the Teen group. Overall, the Teens were better than the Young, but there was general forgetting over time.

##### 3.1.2.2 Reaction time (RT)

Trials with response times beyond 2 SDs for each participant across all conditions were excluded from the RT analyses (Day1 *M* = 1.4 trials, Day8 *M* = 1.0 trials). A repeated measures ANOVA with Time (Day1, Day8) and Condition (Same, Similar) as within factors, and Group (Young, Teen) as between factors revealed main effects of Time (*F(1,46)* = 5.31, *p* = .026, *η_p_^2^* = .10; Day1 *M(SE)* = 2978 (91) ms, Day8 *M(SE)* = 2812 (83) ms), Condition (*F(1,46)* = 88.91, *p* < .001, *η_p_^2^* = .66; Same *M(SE)* = 2717 (80) ms, Similar *M(SE)* = 3073 (84) ms), and Group (*F(1,46)* = 7.20, *p* =  .010, *η_p_^2^* = .14; Young *M(SE)* = 3109 (112) ms, Teen *M(SE)* = 2682 (112) ms). No interaction effects were observed (*p* >  .27). In sum, the Teen group was faster than the Young group, and Same condition trials were responded to faster than the Similar condition trials. Even though there was a decrease in overall accuracy, participants responded faster in general on Day8 than on Day1 for those words whose meanings were remembered (Fig. 2B).

#### 3.1.3 Lexical decision (in the scanner)

##### 3.1.3.1 Accuracy

To reduce the contribution of response bias, behavioral performance was scored as the percentage hit (proportion “word” response to words) minus false alarms (proportion “word” response to pseudowords) for Japanese and Dutch words, separately for both days (Fig. 2C). A repeated measures ANOVA with factors Time (Day1, Day8), Language (Japanese, Dutch) and Group (Young, Teen) revealed main effects of Time (*F(1,46)* = 8.82, *p* =  .005, *η_p_^2^* = .16; Day1 *M(SE)* = 71.8 (2.0) %, Day8 *M(SE)* = 78.7 (1.9) %), Language (*F(1,46)* = 47.78, *p <* .001, *η_p_^2^* = .51; Japanese *M(SE)* = 69.1 (1.9) %, Dutch *M(SE)* = 81.4 (1.8) %), and Group (*F(1,46)* = 33.41, *p* <  .001, *η_p_^2^* = .42; Young *M(SE)* = 65.9 (2.3) %, Teen *M(SE)* = 84.6 (2.3) %), as well as a 3-way interaction Time x Language x Group (*F(1,46)* = 10.01, *p* =  .003, *η_p_^2^* = .18), and a 2-way interaction Time x Language (*F(1,46)* = 31.40, *p* <  .001, *η_p_^2^* = .41). To interpret the 3-way interaction, we ran repeated measures ANOVAs with factors Time and Language, separately for the two groups. For the Young group, a main effect of Language (*F(1,23)* = 15.25, *p* =  .001, *η_p_^2^* = .40) and an interaction effect Time x Language (*F(1,23)* = 31.37, *p* <  .001, *η_p_^2^* = .58) were observed. Posthoc t-tests revealed that participants in the Young group recognized fewer Japanese words on Day1 compared to Dutch words (*t(23)* = 6.36, *p* <  .001), whereas this was not the case on Day8 (*t(23)* = .47, *p* =  .641), as they recognized more Japanese words on Day8 relative to Day1 (*t(23)* = 3.5, *p* =  .002). For the Teen group, main effects of Time (*F(1,23)* = 17.53, *p* <  .001, *η_p_^2^* = .43) and Language (*F* = 34.61, *p* <  .001, *η_p_^2^* = .60) were observed, but the interaction effect was not significant (*F* = 3.843, *p* =  .062, *η_p_^2^* = .143). Participants in the Teen group recognized more Dutch words than Japanese words, and more words on Day8 than on Day1. In summary, lexical decision was better for Dutch words than Japanese words for both groups. More importantly, neither group forgot the trained Japanese words over the course of one week.

##### 3.1.3.2 Reaction time

RT was defined as the time between the word onset and the button response (Fig. 2D). For correctly responded word trials, mean RTs of each condition were analyzed by a repeated measures ANOVA with factors Time (Day1, Day8), Language (Japanese, Dutch) and Group (Young, Teen). This revealed main effects of Time (*F(1,46)* = 10.20, *p* =  .003, *η_p_^2^* = .18; Day1 *M(SE)* = 1890 (51) ms, Day8 *M(SE)* = 1753 (40) ms), Language (*F(1,46)* = 178.99, *p* <  .001, *η_p_^2^* = .80; Japanese *M(SE)* = 2067 (49) ms, Dutch *M(SE)* = 1576 (40) ms) and Group (*F(1,46)* = 10.77, *p* =  .002, *η_p_^2^* = .98; Young: *M*(*SE*) = 1890(51) ms, Teen: *M*(*SE*) = 1753(40) ms), a 3-way interaction (*F(1,46)* = 4.05, *p* =  .050, *η_p_^2^* = .08), two 2-way interactions (Language x Group *F(1,46)* = 6.55, *p* = .014, *η_p_^2^* = .13, Time x Language *F(1,46)* = 28.79, *p* <  .001, *η_p_^2^* = .39).

To unpack the 3-way interaction, repeated measures ANOVAs were conducted separately for the two groups. For each group, the main effect of Language showed that the RT was faster for Dutch than Japanese (Young: Dutch *M*(*SE*) = 1757(68) ms, Japanese *M*(*SE*) = 2154(68) ms, *F(1,23)* = 61.17, *p* <  .001, *η_p_^2^* = .73; Teen: Dutch *M(SE)* = 1395(41) ms, Japanese *M(SE)* = 1980(71) ms, *F(1,23)* = 121.75, *p* <  .001, *η_p_^2^* = .84). For the Young group, main effects of Time (*F(1,23)* = 6.38, *p* =  .019, *η_p_^2^* = .22) as well as an interaction effect between Time and Language (*F(1,23)* = 24.81, *p* < .001, *η_p_^2^* = .52) were observed. This interaction effect was driven mainly by the faster response on Day8 for Japanese words (Day1 *M(SE)* = 2345 (105) ms, Day8 *M(SE)* = 1963 (48) ms, *t(23)* = 4.21, *p* <  .001) which was not the case for Dutch words (Day1 *M(SE)* = 1756 (82) ms, Day8 *M(SE)* = 1758 (75) ms, *t(23)* = .03, *p* =  .98). For the Teen group, the interaction between Time and Language (*F(1,23)* = 6.22, *p* =  .020, *η_p_^2^* = .21) was significant, whereas the main effect of Time just missed the significance threshold (*F(1,23)* = 4.20, *p* =  .052, *η_p_^2^* = .15). The interaction effect was also driven by faster RTs to Japanese words on Day8 than on Day 1 (Day1 *M(SE)* = 2065(76) ms, Day8 *M(SE)* = 1895(81) ms, *t(23)* = 2.51, *p* =  .019) which was not the case for Dutch words (Day1 *M(SE)* = 1393(41) ms, Day8 *M(SE)* = 1397(48) ms, *t(23)* = .14, *p* =  .894).

Overall performance was better and faster for the Teen group compared to the Young group, but both groups improved from Day1 to Day8. Although both groups performed better and faster on Dutch words than Japanese words, their performance on Japanese words became better and faster on Day8 relative to Day1.

#### 3.1.4 Semantic priming

##### 3.1.4.1 Accuracy

Overall accuracy was high (Young: *M_Day1_ =* 93.3(±9.5) %, *M_Day8_ =* 92.6(±5.9) %; Teen *M_Day1_ =* 95.7(±4.0) %, *M_Day8_ =* 95.2(±3.4) %) suggesting that the participants were able to perform the task.

##### 3.1.4.2 Reaction time

Only RTs of correct responses were included in the analysis, and RTs above or below 2 SDs of the participant’s mean were excluded from the analysis (5% for Young, 4% for Teen). Mean RTs of each condition were analyzed by a repeated measures ANOVA with factors Time (Day1, Day8), and Group (Young, Teen) (Fig. 2E). Half of the target words appeared for the first time in the related condition, and the other half of the target words appeared for the first time in the unrelated condition. Due to possible repetition effects, we further included Order (related condition as first, unrelated condition as first) as a factor in the model. This revealed a main effect of Time (*F(1,46)* = 5.43, *p* =  .024, *η_p_^2^* = .11; Day1 *M(SE)* = -27.6 (8.5) ms, Day8 *M(SE)* = .20 (8.1) ms, and a trend in the 2-way interaction Time x Group (*F(1,46)* = 3.71, *p* =  .06, *η_p_^2^* = .08). No significant main effect of Order (*p* = .307) or any interaction effect with factor Order was found (*p* >  .196). The interaction effect Time x Group was driven by the trend for the Young group to have a larger difference in the priming effect between the two sessions compared to the Teen group (*t(28.1)* = 1.93, *p =* .064, *M_diff_Young_* = 51 (111) ms, *M_diff_Teen_* = 5 (37) ms).

To see whether there were any priming effects, we performed a one-sample t-tests on the priming effect (RT difference unrelated – related) averaged across the two order conditions, separately for Days and Group (adjusted alpha = .0125). For the Young group, RT on Day 1 was larger for the related than unrelated (*t(23)* = 2.62, *p* = .015, difference RT *M(SD)* = -42(79) ms), but this difference was not observed on Day8 (*t(23)* = .56, *p* =0.58, *M(SD)* = 8(75) ms). For the Teen group, similar results were observed (Day 1 (*t(23)* = 2.31, *p* = .031, *M(SD)* = -13(27) ms; Day8 (*t(23)* = 1.51, *p* =0.145, *M(SD)* = -8(26) ms).

#### 3.1.5 Effect of individual verbal/non-verbal skills on memory performance

Working memory span is known to affect vocabulary development in children (e.g., Gathercole and Adams, 1994; Gathercole and Baddeley, 1989). Moreover, word learning studies have shown that vocabulary size affects learning (James et al., 2019) and consolidation (James et al., 2017) of novel words. We could expect consolidation/integration trajectories to differ between individuals with different working memory spans and vocabulary sizes. As exploratory analyses, we ran separate analyses for the above tasks (free recall, association test, lexical decision and semantic priming) with the behavioral test results (PPVT, Digit span, Ravens) as covariates. These analyses revealed that participants with higher PPVT scores (thus, bigger vocabulary size) were in general good at learning novel words (association test accuracy; *F(1,43)* = 4.48, *p* =  .040, *η_p_^2^* = .094), and showed a trend for better free recall on Day8 compared to Day1 (*F(1,41)* = 3.16*, p* = .083, *η_p_^2^* = .072). Digit span, in contrast, interacted with change in lexical decision accuracy across the two sessions (*F(1,43)* = 9.12, *p* =  .004, *η_p_^2^* = .175), change in reaction time for lexical decision across the two sessions (*F(1,43)* = 6.99, *p* =  .011, *η_p_^2^* = .140), as well as semantic priming effect (*F(1,43)* = 4.55, *p* =  .039, *η_p_^2^* = .096). In general, participants with lower digit span improved more on their performance on Day8. However, free recall performance overall was better for the children with longer digit span (*F(1,41)* = 5.19, *p* =  .028, *η_p_^2^* = .112). Full reports of these analyses can be found in the Supplementary material 6.

#### 3.1.6 Summary: behavioral results

Both free recall and lexical decision performance on Japanese words showed that children remembered more words on Day8 than on Day1. Performance on the association memory task, however, was better on Day1 than on Day8 although for the correct responses, children became faster at responding on Day8. Moreover, the Young group’s performance was worse than that of the Teens for pictures that were different from the ones presented during training. The semantic priming data showed a negative priming effect on Day1, and no priming effect on Day 8, for both groups. Participants with larger vocabulary size showed better word-meaning association learning and better retention of the word-forms. Participants with longer digit span showed better word-form memory and a reduction in negative priming effect over time.

### 3.2 Imaging

We asked whether the neural activity pattern for Japanese words differed from that of Dutch words, and whether the activity pattern changed as a function of time. We expected more activation in the hippocampus on Day1, and a decrease in the difference between the Japanese and Dutch words on Day8. Furthermore, we asked whether the results would differ for the two age groups. Given that prior behavioral studies showed a similar performance change with time for young adults and children, we predicted that critical regions (hippocampus, left pMTG) would not show age-related differences.

To answer the questions regarding the language difference and change over time, we compared the contrast images of the conditions of interest using a flexible factorial design with factors Time (Day1, Day8), Language (Japanese, Dutch) and Subjects (each participant received a regressor).

#### 3.2.1 Main effect of language: Japanese vs Dutch

**Fig. 3:**
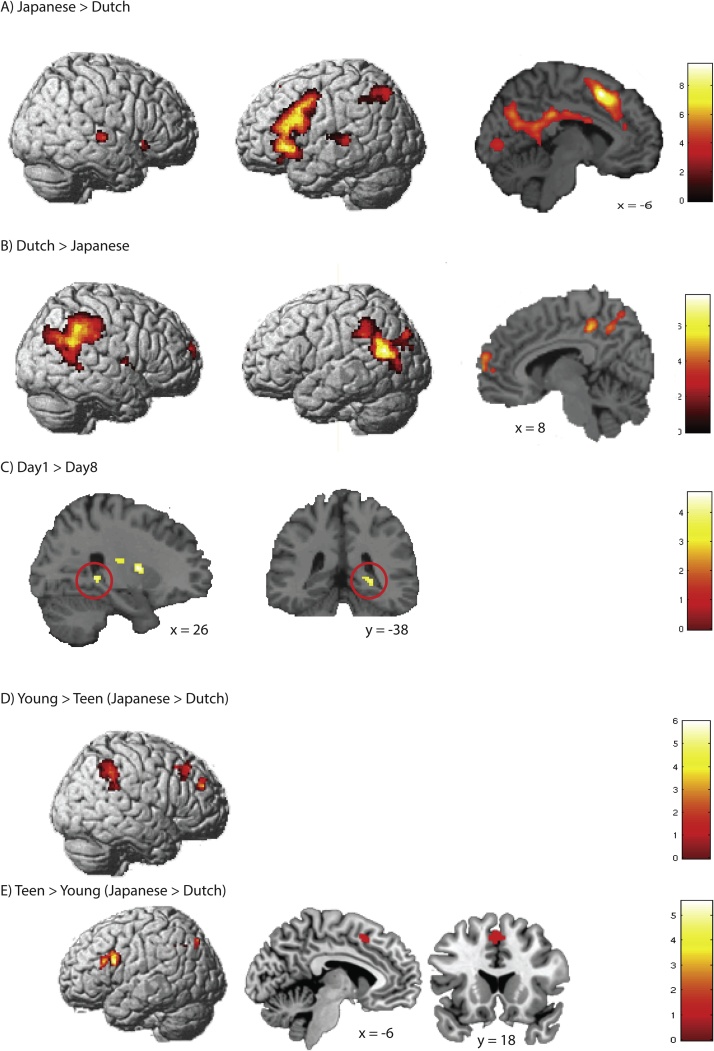
*Imaging results*. A) Areas where activation was greater during Japanese word recognition than Dutch word recognition. B) Areas where activation was greater during Dutch word recognition than Japanese word recognition. C) An area within the right hippocampus decreased in activity over time (hippocampus area circled in red). Clusters were significant (family-error wise corrected within the hippocampal region of interest) at the cluster-level inference of p < 0.05 with a voxel-level t.hresholded at p < 0.001, uncorrected, rendered on a template brain, and overlaid on template sagittal and coronal slices. D) Contrast Japanese vs Dutch was compared between the Young > Teen groups. E) Contrast Japanese vs Dutch was compared between the Young < Teen groups and F) Teen > Young groups. Clusters that showed a significant difference between the groups are superimposed onto a template brain.

Comparing the activation of Japanese words to Dutch words across Day1 and Day8 collapsed across the two groups, we observed an activation increase in distributed areas of the brain (Fig. 3A and Supplementary Table 3). Specifically, areas in the left supplementary motor area, bilateral insula extending to pars triangularis and opercularis of the inferior frontal gyrus (IFG), left middle part of the middle/superior temporal gyrus (MTG/STG), right superior temporal gyrus (STG), left posterior cingulate cortex, left inferior parietal lobe (IPL) and left calcarine gyrus were more activated for Japanese relative to the Dutch words. The reverse effect (Dutch > Japanese) showed an activation increase in areas in the posterior temporal/parietal cortex as well as the midline structures in the posterior cingulate cortex and middle frontal gyrus (Fig. 3B). Thus, Dutch words relative to Japanese words elicited heightened activation in areas known to code for semantic information (Binder et al., 2009; Price, 2012). The Japanese words elicited more areas in the STG, and fronto-parietal attentional network encompassing the IPL and left IFG/premotor cortex – probably due to the fact that the participants needed more attention in order to perceive the auditory stimulus in a noisy environment (Buschman and Miller, 2007; Shinn-Cunningham, 2008).

#### 3.2.2 Main effect of time: Day1 vs Day8

The general change over time on the whole brain level showed no significant clusters. Since we had an a priori hypothesis that the hippocampus would be more involved right after learning the new words, but that with time the involvement of the left pMTG would increase, we looked specifically into these two regions for a change over time. Activation in the right hippocampus decreased with Time (small volume corrected (SVC) *p_FWE_* = 0.032, k = 27, peak coordinate Montreal Neurological Institute (MNI) [26–38 0]). No areas showed an increase with Time, not even within the restricted pMTG region of interest (ROI).

We did not observe significant Time x Language interaction effects. When looking at Day1 vs Day 8 changes in activation patterns in the hippocampus and left pMTG for each language separately, we found a trend for decrease in the right hippocampus cluster for Japanese words (SVC *p_FWE_* = 0.080, peak MNI [28–40 2], k = 13) overlapping to the cluster observed for Day1 > Day8 comparison. We did not observe an increased activation in the left pMTG for Japanese words on Day8 relative to Day1. As expected, Dutch words did not show any significant increases or decreases with time.

Behaviorally, the size of the priming effect varied across participants, especially for the Young group. We expected more involvement of the left pMTG with integration of novel words, which would be paralleled by a greater priming effect in the behavioral data. Focusing on the activation pattern for the Japanese condition, we tested whether there was a positive correlation between brain activation patterns and the semantic priming effects that changed from Day1 to Day8. To test this hypothesis, we conducted a general linear model with the contrast between Day1 and Day8 for the Japanese condition as one regressor, and difference in priming effects (Day1-Day8) as another regressor. For the whole brain analysis, a positive correlation was found in a cluster that included the left pre- and post- central gyri. Negative correlations were found in the bilateral superior and middle frontal gyrus. When restricting the search area to the left pMTG ROI, we did not find any significant correlations.

#### 3.2.3 Group differences

Next, we asked whether the brain activation pattern differed between the Young and Teen groups. First, we compared the activity difference between the Japanese and the Dutch words pooled across Day1 and Day8, and compared the two participant groups. This comparison showed that when processing Japanese words relative to Dutch words, the Young group showed greater activation in the right inferior parietal lobes/supramarginal gyrus, and right middle frontal gyrus, whereas the Teens showed greater activation in the left supplementary motor area/ inferior frontal gyrus (pars triangularis/opercularis) areas (Fig. 3DE, Supplementary Table 4).

When comparing the activation patterns for Japanese relative to Pseudo-Japanese condition, we did not see any group differences. Change over time for the Japanese words (Japanese Day1 vs Day8) also did not show any significant group differences.

#### 3.2.4 Summary: imaging results

Overall, novel Japanese words evoked activation in the fronto-parietal attentional areas compared to Dutch words. Dutch words, in contrast, showed activation increases in the posterior temporal and parietal areas, known to be activated for semantic retrieval (Binder et al., 2009; Price, 2012). Regarding activation change with time, we observed a decrease in activation in the right posterior hippocampus, which seems to have been mainly driven by the Japanese condition. Against our predictions, but in line with the lack of a behavioral priming effect regarding the integration of novel words, we did not observe an increase in activation in the left pMTG with time for the Japanese condition. A between-group comparison for the Japanese condition showed greater right hemisphere activation for the Young group, whereas the Teen group showed greater activation in the left prefrontal cortex.

## 4 Discussion

The standard systems-level memory consolidation theory posits an initial memory representation in the hippocampal network, which shifts towards a neocortical network with consolidation (Alvarez and Squire, 1994; Frankland and Bontempi, 2005). Previous studies testing young adults have shown that representations of novel words also undergo such changes with consolidation, with greater involvement of the hippocampus just after learning (Davis et al., 2009), and an increased involvement of the left posterior middle temporal gyrus (pMTG) after a time of offline consolidation (Bakker-Marshall et al., 2018; Takashima et al., 2014, 2017). Here, we tested whether children would demonstrate a similar pattern. We further asked whether there would be differences between 8- to 10-year-olds and 14- to 16-year-olds (approximately before and after puberty), as children’s brains are still undergoing structural changes during this period (Herting and Sowell, 2017).

Behavioral results showed that children were able to learn novel words in one training session and retain the words over the course of one week. Although the meanings/referents of the words were less well remembered after a week, memory for the word forms was better on Day8 than on Day1, probably due to the children being exposed to the word form during multiple tests. Unlike prior adult data, however, the lexicalization (integration) effect, indexed by the semantic priming effect, did not show the expected pattern. Whereas young adults showed no priming effect immediately after training, but this effect emerged after a time delay of at least a day (Bakker et al., 2015a, 2015b), the children in this study showed a negative priming effect on Day1 and no priming effect on Day8.

In line with our previous adult data, the imaging results showed that hippocampal activity decreased with time, especially for the Japanese words. We did not observe, however, any other changes as a function of time. Specifically, we did not observe an activation increase in the left pMTG for the trained novel words, as we had observed in prior studies testing young adults (Takashima et al., 2014, 2017). The activation level was greater for the Dutch words relative to Japanese words in the left pMTG for both testing sessions. The behavioral and neural data jointly suggest that one training session and one week of offline consolidation may not have been enough for the novel words to be represented like existing familiar words in the children’s native language.

Past studies on word learning in school-aged children have shown that behavioral integration effects already appear after a night of consolidation in the form of word-form competition effects (Henderson et al., 2015, 2012, 2013a; Henderson et al., 2013b) and semantic priming (van der Ven et al., 2017). We did not observe such changes even after a week, but rather a negative priming effect on the day of training, which disappeared in the second session. Previous cross-linguistic semantic priming studies have shown positive priming effects, albeit with mixed results (reviewed in van Hell and Kroll, 2012). L1-L2 usually shows greater priming effects than L2-L1, and longer stimulus onset asynchrony (SOA) (Lee et al., 2018) and larger item set sizes are additional factors that contribute to larger priming effects (Wen and van Heuven, 2017). We were limited in the number of items tested due to the small number of trained words and the difficulty in creating a set of semantically related target words with multiple restrictions (i.e., the target words should be known to the 8-year-olds and not used in the description of the trained word). Nonetheless we tried to maximize the semantic activation of the prime word by having a long SOA of 250 ms. The imaging results also did not confirm our hypothesis that word representations would emerge in the left pMTG, as we had observed in young adults (Bakker-Marshall et al., 2018; Takashima et al., 2014, 2017). Although a study on novel word learning by Landi et al. (2018) showed increased left STG/MTG, precuneus and IPL activation after a 24-hour consolidation period in adolescents and younger adults, to the best of our knowledge, there is no prior word consolidation fMRI data on younger children. It is therefore difficult to pin-point the exact factors that underlie the discrepancy between the young adult data and the present study, but a few speculations can be made.

One possibility is that we provided an inadequate amount of time and practice for the novel word representations to consolidate and integrate. Although we delayed our second session by a week rather than testing after only a 24-hour delay, we did not capture evidence of activation increases over time in any neocortical areas. The meanings of some of the words were forgotten with time (as shown by performance on the association memory test). This suggests that one training session may not have been enough for word meanings to be retained and integrated over a period of one week, even though word-form memory (tested in the free recall test and the lexical decision task in the scanner) was still intact at delayed test. If we had tested integration on the word-form level, for example with a pause detection task (e.g., Bakker et al., 2014; Gaskell and Dumay, 2003; Henderson et al., 2013a), we might have observed an integration effect. However, due to the nature of the novel words, it was not possible to find words in Dutch that were phonological neighbors of the trained Japanese words. Since the semantic priming effect relies on the strength of the prime word to activate the semantically neighboring words in the target language, our training protocol may not have been long enough to create a strong semantic representation of the novel word. Many previous studies on word consolidation in children have used up to 20 words in training. However, it was necessary for our fMRI paradigm to have as many trials as possible to model brain responses to trained novel words, and we therefore opted for 30 words in the training set.

Another possibility is that the maturation of the semantic network in the neocortex may still be under construction for the children compared to young adults. Schema memory research proposes faster assimilation of new information if there is an existing schema or prior knowledge related to the novel information (Bartlett, 1932; Tse et al., 2008, 2007; van Kesteren et al., 2014, 2012). Adults may have more well-established semantic schemata than children, and thus the pMTG may be more developed in adults than in children, facilitating the integration of novel words. However, this explanation is challenged by the fact that we did not see a difference between the Young and the Teen groups in terms of changes in pMTG activation levels on Day1 versus Day8. Moreover, behavioral studies showing integration effects after 24 h in children of the same age as those in our younger age group (Henderson et al., 2015, 2012, 2013a; Henderson et al., 2013b; Van der Ven et al., 2017) further challenge the plausibility of this explanation.

A third possible explanation pertains to the unfamiliarity of the novel words and their concepts in our study. Prior studies have used stimuli that were part of the participants’ native language (Henderson et al., 2013b; van der Ven et al., 2017), or a set of trained pseudowords that sounded similar to native words (Henderson et al., 2012, 2013a). Taking the schema advantage for memory consolidation into consideration (van Kesteren et al., 2014, 2012), words that conform to learners’ native language or concepts that are familiar may be easier to integrate. A recent review supports this suggestion: Children with a richer vocabulary show better word learning (James et al., 2017). Our study also found that children with higher vocabulary scores showed better word-meaning association learning. Furthermore, a recent study showed that 7- to 9-year-old children recall new words better immediately after learning if those words have many phonological neighbors than if they have no neighbors (but this difference disappears on recall a week after learning suggesting better consolidation of the no-neighbor words; James et al., 2019). The children in our study could therefore have made use of their native language knowledge to integrate the novel native language words, but this integration process may take longer, or more repeated exposure may be necessary for foreign words to be integrated. Both the phonological structure and the concepts were new to the participants, making it harder for novel words to be integrated into the existing native language vocabulary network. The newly created representations could even have been kept apart in a separate network, thereby further reducing the chance of integration. Some researchers even claim that the second language (L2) lexicon is stored separately from the native lexicon (L1), which would predict no interaction effects between L2 and L1 words (Qiao and Forster, 2017). The differential brain activity patterns for Dutch words and Japanese words clearly show that the temporo-parietal areas including the pMTG were more active for the familiar Dutch words, whereas for Japanese words, activity in the IFG and insula as well as the perception-related superior/middle temporal and calcarine areas were more prominent. This suggests that more effortful search-related and attention-related processes were involved during the processing of Japanese words. Slower and less accurate performance on Japanese items compared to Dutch items further supports the interpretation that the processing of novel words was more effortful than that of familiar Dutch words.

We opted to train participants with unfamiliar concepts to circumvent issues arising from translation asymmetries, that is, stronger word-concept links for L1 compared to L2 leading to smaller or non-observable L2 to L1 priming (e.g. Sabourin et al., 2013). We hoped to observe novel word learning that would be similar to L1 novel word learning (novel word to novel concept) and expected a similar behavioral semantic priming effect to that found in a study using L1 novel words in school-aged children (van der Ven et al., 2017). Furthermore, we used a long SOA (250 ms) so that the participants would have enough time to process the novel word (Lee et al., 2018). Nonetheless, the novel words and/or novel concepts were not able to pre-activate the target word enough for semantic priming to be observed. Rather, activation of the novel concept through hearing the novel word might have interfered with the processing of the target word when it was semantically related, leading to a negative priming effect on Day1. Negative priming effects are often found when there is a conflict in response options between the current and the previous stimulus (Fox, 1995; Tipper, 1985). However, it is difficult to interpret the negative priming effect from this point of view as we did not have response conflict in our paradigm. It is more likely that the novel word representations were too weak to produce semantic priming, especially given that we did not observe activation patterns in the semantic/lexical areas in the pMTG to novel trained words on Day8.

Regarding the Young and Teen group differences, we observed an interesting lateralization difference. The activation pattern contrast for Japanese versus Dutch words was more right lateralized for the Young compared to the Teens, whereas the Teens activated the left IFG more than the Young. Although some studies have shown a left-lateralization of language processing already in neonates and infants (Dehaene-Lambertz et al., 2002; Peña et al., 2003), other studies measuring brain activity in school-aged children have shown a shift from right to left lateralization with increasing lexical knowledge or familiarity of the words (Sugiura et al., 2011; Szaflarski et al., 2006). According to Holland and colleagues’ study of 7- to 18-year-olds, left lateralization increased with age when doing a verb generation task in the scanner (Holland et al., 2001; reviewed in Vannest et al., 2009). We observed a higher left IFG activation pattern for the Teens relative to the Young during lexical decision on the trained Japanese words. This area may become more involved in word retrieval as the size of lexicon increases with age. It has been shown that earlier sensory regions mature before the higher-order association regions of the brain (Gogtay et al., 2004; Shaw et al., 2008). Cognitive maturation may be paralleled by brain maturation and specialization in neural development.

We may have failed to observe neural effects due to the quality of the imaging data, especially in the Young group. Although we made the scanning session as comfortable as possible and secured their heads with pads to stabilize in the head coil, children, especially the younger ones, had a hard time keeping still in the scanner for 15 min. We further employed an extensive preprocessing procedure to remove movement related artifacts from the imaging data, but we still had to remove 5 data sets from the Young group due to excessive movements resulting in fewer data points for this group. Repetition of procedure helped improve the quality of the data and Day8 recordings contained fewer movement-related artifacts than Day1 recordings, which may have affected statistical sensitivity for detecting between-session effects.

## 5 Conclusion

In this study, we trained school-aged children on novel Japanese words that referred to unfamiliar objects, and measured their brain responses while they were recognizing the words in the MRI scanner at two time points, once just after training and once after a week. Partially in line with the systems-level consolidation theory, hippocampal activation decreased after a week. The expected activation increase of the left posterior middle temporal gyrus with time, as well as a lexical integration effect measured with the semantic priming task, however, were not observed. This may be due to the novel words and concepts being relatively unfamiliar to the participants, delaying the course of integration compared to earlier studies that used native(-like) words as stimuli. We observed a shift from right to left hemisphere involvement between the primary school-aged children and secondary school-aged children, which is likely due to maturation of the language network.

## Declaration of interest

The authors have declared that no competing interests exist.
