# Supplementary material for: Neural correlates of word learning in children
Source: Dev Cogn Neurosci. 2019 Apr 28;37:100649. doi: 10.1016/j.dcn.2019.100649 (PMC6969309; doi:10.1016/j.dcn.2019.100649)
Supplement: Supplementary file 1 [file mmc1.docx]

# Supplementary Table 1.

Participant demographics

|  | **Young group** | | | **Teen group** | | |
| --- | --- | --- | --- | --- | --- | --- |
|  | ave | std | range | ave | std | range |
| **Age (years; month)** | 9;9 | 9.7 m | 8;6 -10;11 | 15;6 | 10.9 m | 14;1-16;11 |
| **Gender Male/Female** | 7/17 |  |  | 8/16 |  |  |
| **Digit span forward** | 7.9 | 1.4 | 5-11 | 9.4 | 2.2 | 6-13 |
| **Digit span backward** | 5.3 | 1.6 | 2-8 | 6.8 | 1.6 | 4-12 |
| **Digit span normalized** | 11.6 | 3.0 | 6-16 | 10.9 | 2.7 | 6-16 |
| **Word span** | 5.8 | 1.2 | 4-8 | 7.3 | 1.5 | 5-10 |
| **PPVT score normalized** | 106.8 | 12.1 | 78-127 | 106.1 | 11.5 | 85-125 |
| **Raven score percentile** | 80.0 | 19.4 | 30-100 | 69.6 | 28.6 | 12-100 |

Ave: average, std: standard deviation, PPVT: Peabody Picture naming Vocabulary Test

# Supplementary Table 2

A list of trained Japanese words, pictures, definitions, and target Dutch words used in the semantic priming task

| Japanese word | picture 1 | picture 2 | definition Dutch (long) | definition Dutch (short) | | | Related Dutch word 1 | | Related Dutch word 2 | | Pseudo 1 | | Pseudo 2 |
| --- | --- | --- | --- | --- | --- | --- | --- | --- | --- | --- | --- | --- | --- |
| torii | 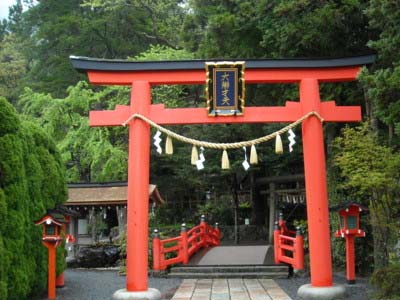 | 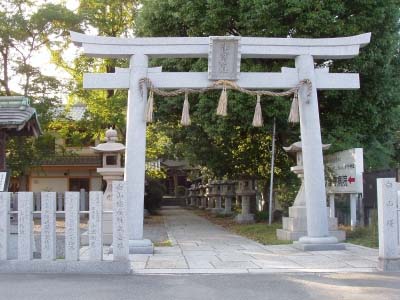 | Een grote, open poort die toegang geeft tot Japanse heiligdommen. | | open poort voor Japanse heiligdommen | | entree | | ingang | | engrij | | inbalt |
| shouji | 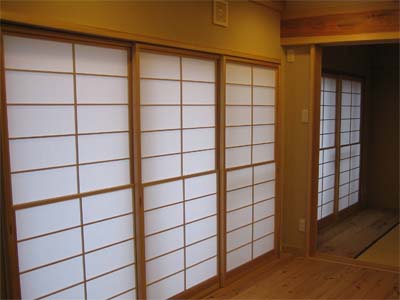 | 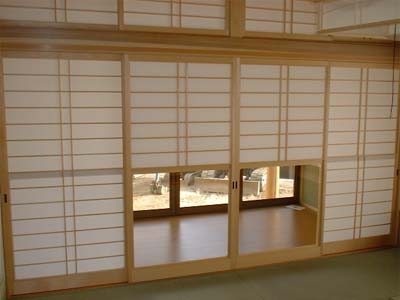 | Een schuifdeur gemaakt van hout bestaande uit met touw vast geweven horizontale plankjes bamboe of hout. | | schuifdeur met hout en papier | | muur | | wand | | muwt | | wamp |
| sudare | 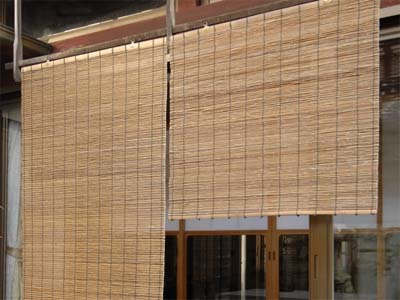 | 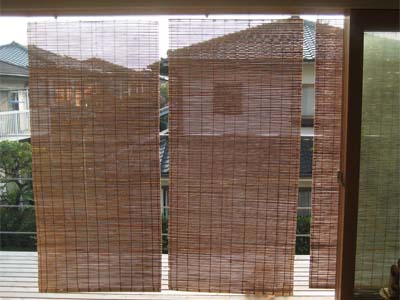 | Een geweven scherm dat in ramen en deuropeningen gehangen wordt, bestaande uit met touw vast geweven horizontale plankjes bamboe of hout. | | geweven scherm met bamboe of hout | | gordijn | | venster | | gokkijn | | benstes |
| yoroi | 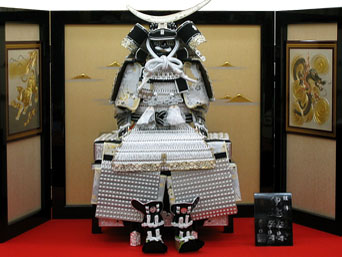 | 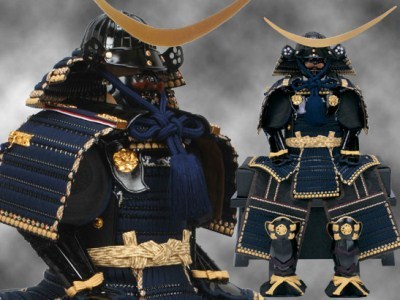 | Flexibel harnas gemaakt van meerdere lagen leer, staal en koper. | | flexibel harnas | | schild | | ridder | | schirn | | ludder |
| zaisu | 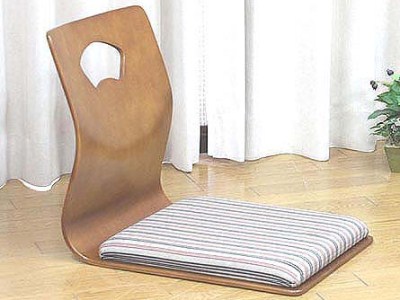 | 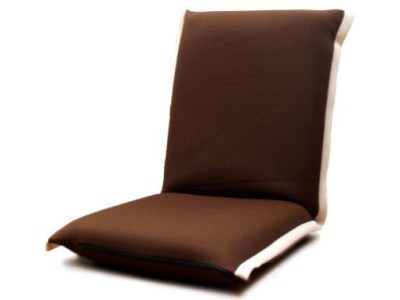 | Traditionele Japanse stoel zonder poten om mee op de grond te zitten. | | stoel zonder poten | | leuning | | meubel | | reuging | | weibel |
| magonote | 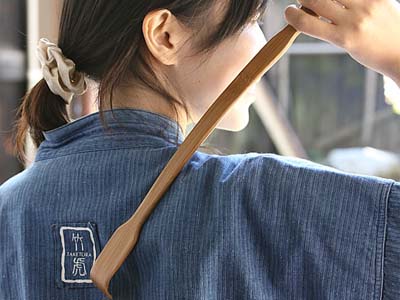 | 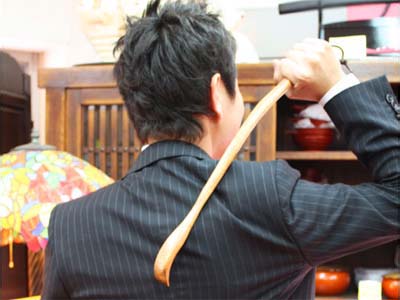 | Een stok om op je rug te kunnen kriebelen op plaatsen waar je met je handen niet bij kan. | | stok om je rug te kriebelen | | jeuk | | krabber | | jijk | | spibber |
| renge | 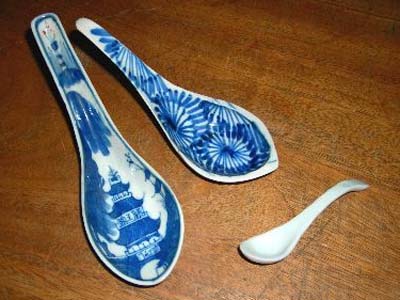 | 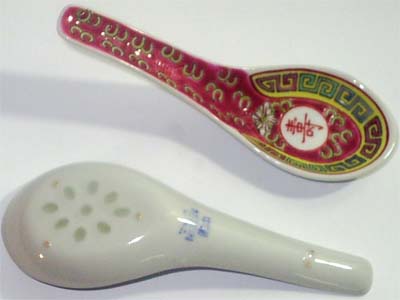 | Een korte porseleinen lepel met een breed handvat, typisch Aziatisch. | | porseleinen lepel | | mes | | vork | | pes | | vops |
| hyotan | 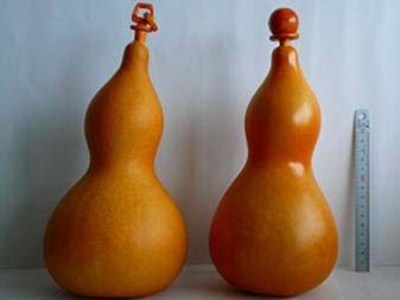 | 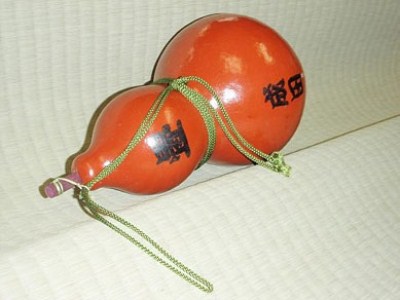 | Kruik die gebruikt wordt om water, wijn of kruiden in te bewaren, gemaakt uit een soort pompoen. | | kruik van pompoen | | drank | | fles | | grank | | zwes |
| wasabi | 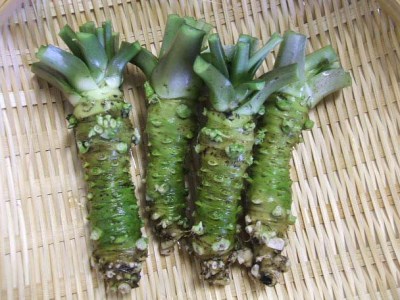 | 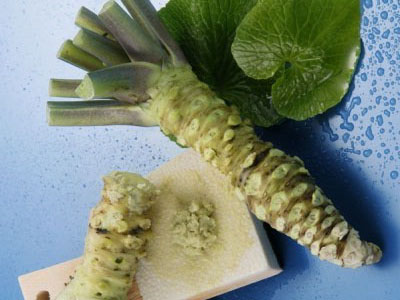 | Heel erg heet smakende Japanse plant met een opvallende groene kleur. | | groene hete Japanse plant | | pittig | | scherp | | postig | | schebs |
| youkan | 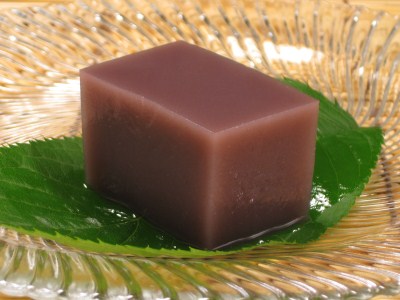 | 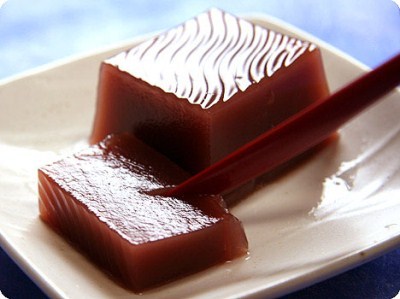 | Zoet Japans nagerecht dat gemaakt is van rode bonen, gelei en suiker. | | zoet nagerecht met bonen | | pudding | | toetje | | purking | | moespe |
| chikuwa | 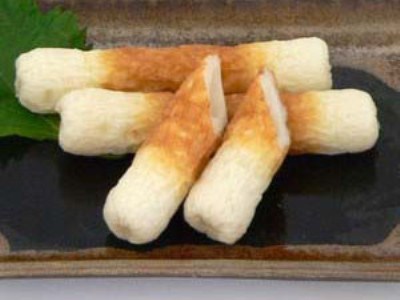 | 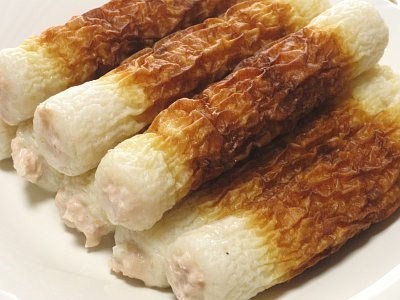 | Een Japanse snack in de vorm van een hol buisje, gemaakt met onder andere gepureerde kabeljauw. | | snack met gepureede kabeljauw | | visstick | | loempia | | viskleck | | leirvia |
| ema | 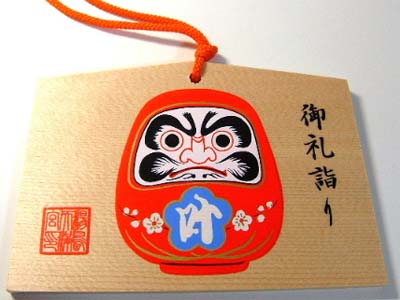 | 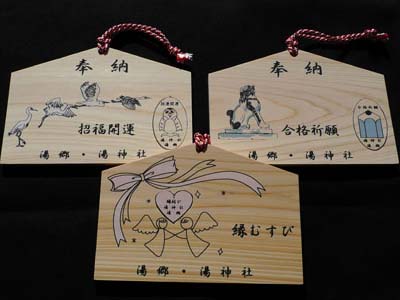 | Een houten plankje waarop je een verlanglijstje schrijft, je hangt ze daarna op bij een tempel. | | houten plankje voor verlanglijstje | | wens | | gebed | | wemt | | gegad |
| karuta | 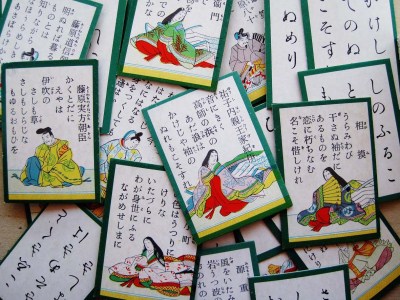 | 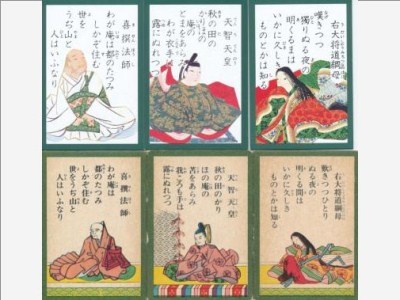 | Spel waarbij de eerste helft van een gedicht wordt voorgelezen en je de tweede helft van het gedicht moet zoeken. | | spel met gedichten | | rijm | | kaarten | | kijm | | kijnten |
| mikuji |  | 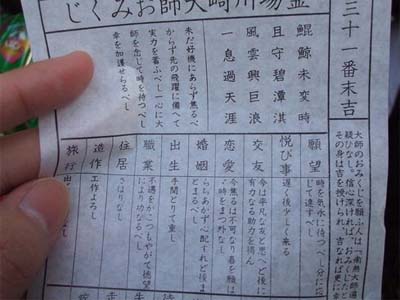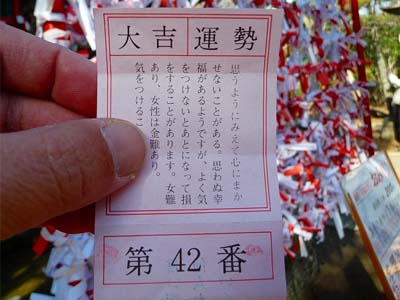 | Papiertje dat je in een tempel kan krijgen met een voorspelling van je toekomst. | | papiertje met je toekomst | | lot | | horoscoop | | lon | | horokwijf |
| tourou | 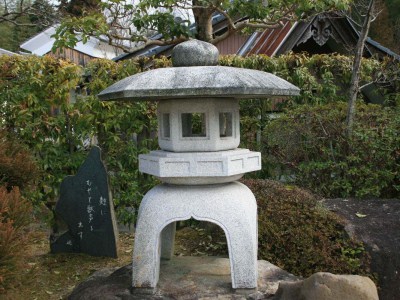 | 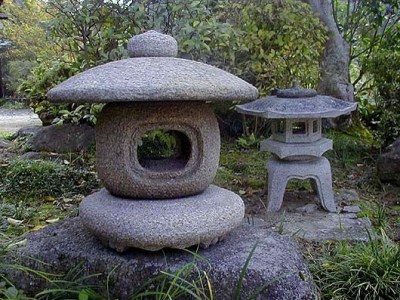 | Ouderwetse Japanse lantaarn die je buiten tegenkomt bij tempels en begraafplaatsen. | | ouderwetse lantaarn | | lamp | | tuin | | lals | | tean |
| kappa | 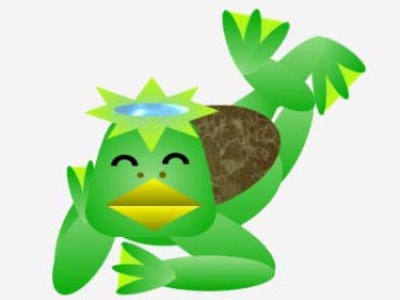 | 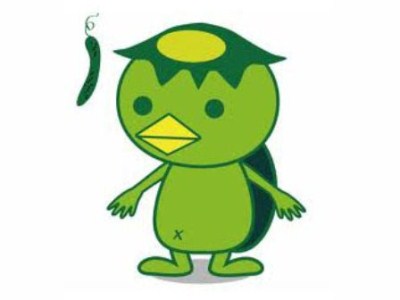 | Klein Japans sprookjesdier dat in rivieren woont, hij is groen en heeft zwemvliezen. | | groen sprookjesdier | | kikker | | snavel | | hukker | | vlazel |
| tanuki | 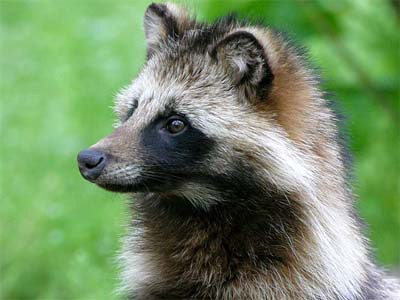 | 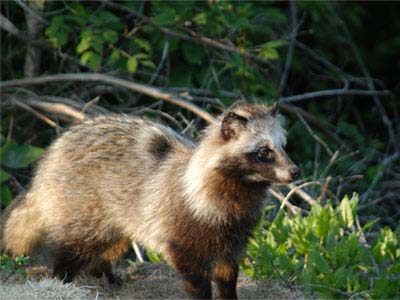 | Een soort kleine das die er in Japanse fabels om bekendstaat iedereen voor de gek te houden. | | kleine das | | wasbeer | | vos | | walteer | | wos |
| suzuri | 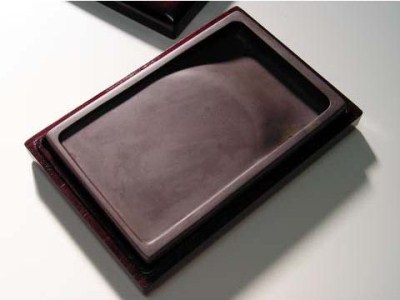 | 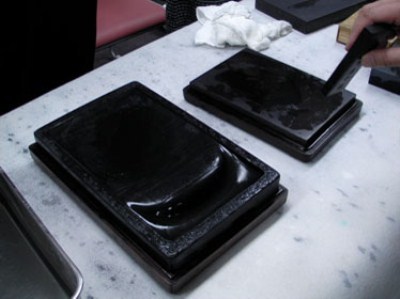 | Stenen bakje om inkt in fijn te malen en te bewaren. | | stenen bakje voor inkt | | stempel | | pen | | stetjel | | peb |
| shamisen | 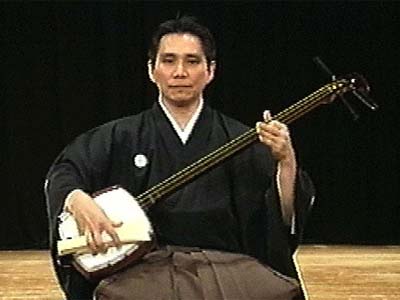 | 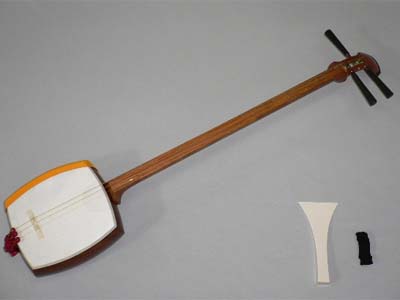 | Een traditioneel Japans muziekinstrument met drie snaren. | | muziekinstrument met drie snaren | | viool | | gitaar | | biael | | gutoer |
| gokihoi | 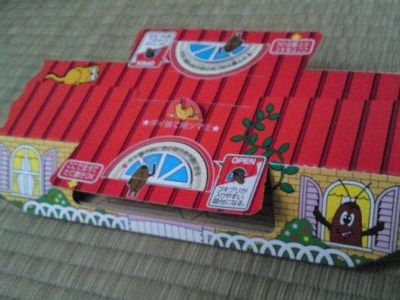 | 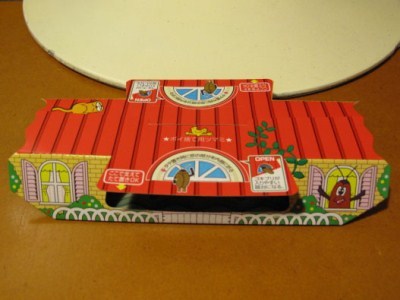 | Een klein kartonnen huisje met een kleverige bodem om kakkerlakken in te vangen. | | klein kartonnen huisje voor kakkerlakken | | mieren | | val | | kuiren | | jal |
| senko | 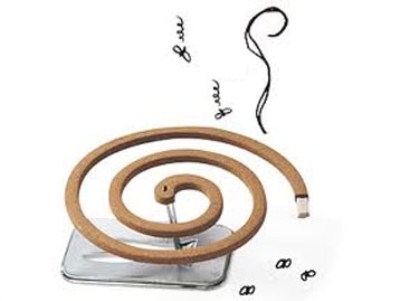 | 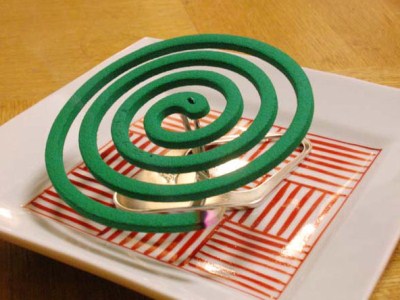 | Wierook die speciaal bedoeld is om muggen en andere insecten weg te jagen. | | wierook tegen muggen | | spiraal | | rook | | kridaal | | roor |
| obi | 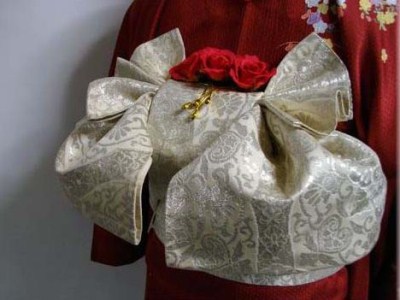 | 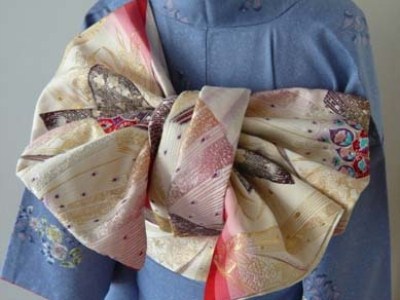 | Een lint voor het dichthouden van traditionele Japanse kleren vaak met een mooie knoop. | | lint voor Japanse kleren | | riem | | strik | | diem | | tsjik |
| geta | 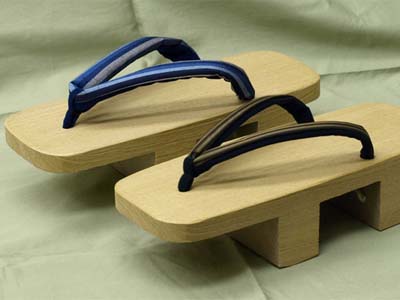 | 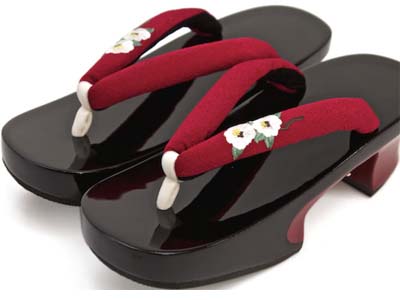 | Houten Japanse sandalen die iets verhoogd zijn zodat je geen modder aan je voeten krijgt. | | verhoogde sandalen | | slipper | | schoen | | spepper | | schoer |
| kanzashi | 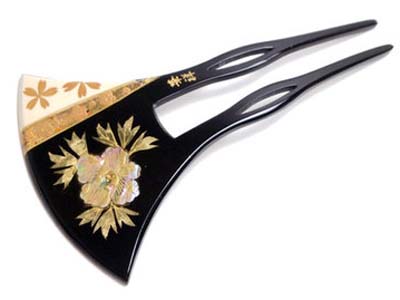 | 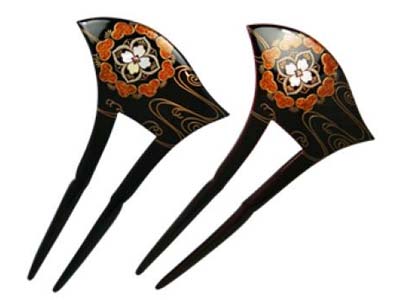 | Beschilderde houten haarversiering om in een knot te steken. | | houten haarversiering | | kapsel | | pruik | | kousel | | priek |
| shiro | 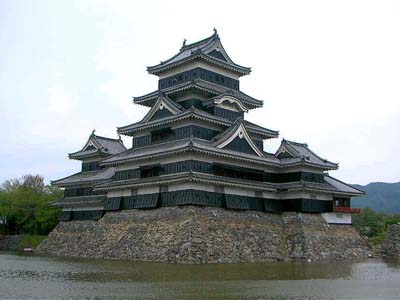 | 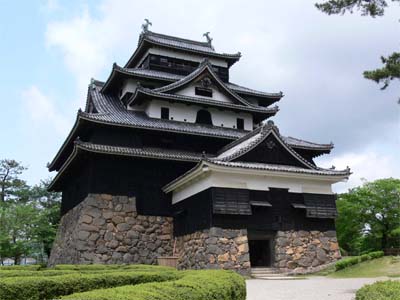 | Middelste toren van een Japans kasteel. | | Japans kasteel | | paleis | | daken | | paroos | | hapen |
| irori | 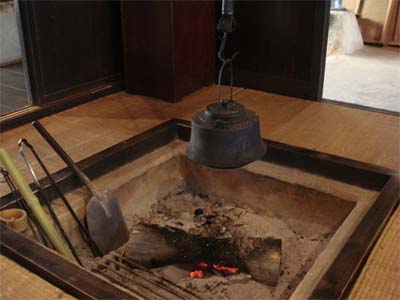 | 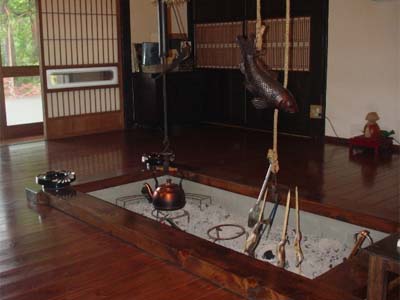 | Een kookplaats die in de grond zit en naast koken ook wordt gebruikt om het huis mee te verwarmen. | | kookplaats in de grond | | oven | | kachel | | aben | | maspel |
| happi | 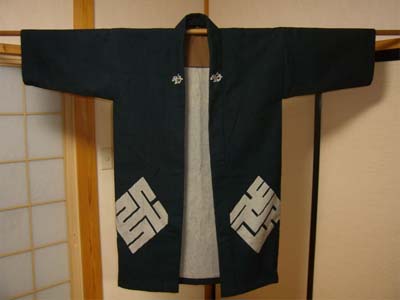 | 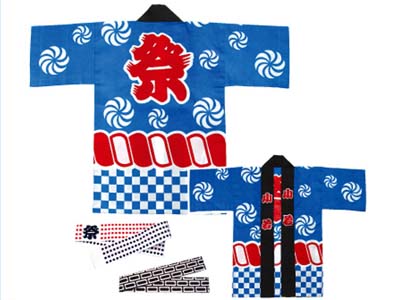 | Japanse traditionele kleding met rechte mouwen voor feestdagen en speciale gelegenheden. | | kleding met rechte mouwen | | jas | | vest | | zas | | vekt |
| kadomatsu | 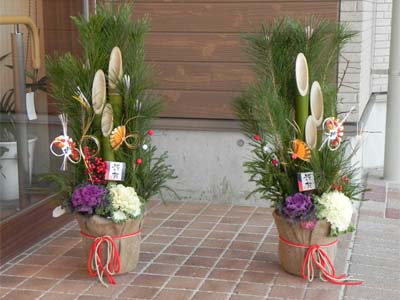 | 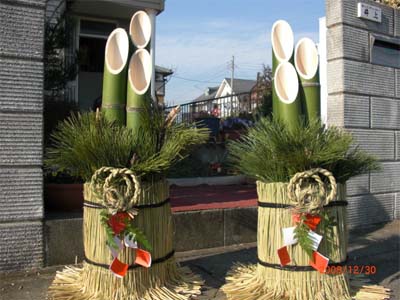 | Een traditionele Japanse versiering voor nieuwjaar waarvan je er twee bij de deur zet. | | versiering voor nieuwjaar | | kerststukje | | bloempot | | dindsstukje | | ploemput |
| hagoita | 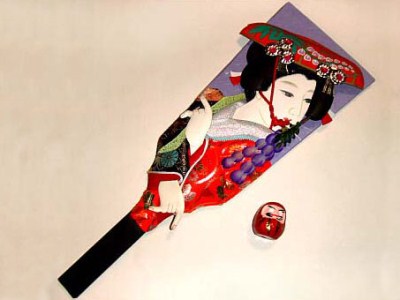 | 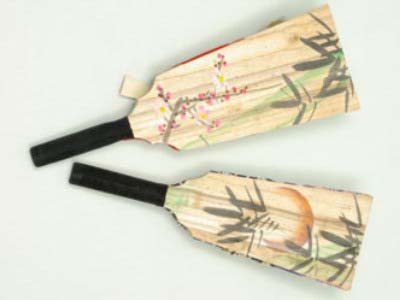 | Rechthoekige houten rackets om balspellen mee te spelen die vaak versierd zijn met een schilderij. | | versierde houten rackets | | tennis | | pingpong | | penbis | | pergpong |
| pachinko | 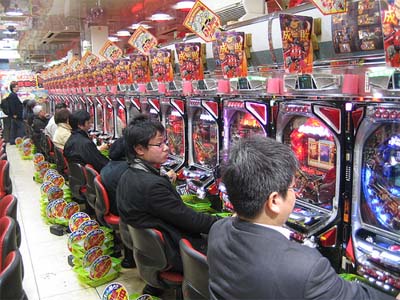 | 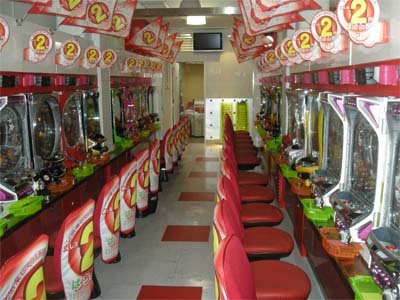 | Een soort Japanse rechtopstaande flipperkast waarop je om geld kan spelen. | | rechtopstaande flipperkast | casino | | gokken | | faciko | | vikken | |

# **Supplementary Table 3.** Main effects of language

| cluster | | | | peak | | | | |
| --- | --- | --- | --- | --- | --- | --- | --- | --- |
| p(FWE-corr) | size | | Z-value | | x | y | z | AAL location |
| **Japanese > Dutch** | | |  | |  |  |  |  |
| 0 | 1597 | | 0 | | -6 | 16 | 52 | Supplementary motor area |
|  |  | | 4.76 | | -10 | 28 | 28 | left anterior cingulate |
|  |  | | 3.99 | | 10 | 32 | 30 | right middle cingulum |
|  |  | |  | |  |  |  |  |
| 0 | 3402 | | 6.97 | | -30 | 22 | 2 | left insula |
|  |  | | 6.88 | | -46 | 8 | 38 | left precentral |
|  |  | | 6.66 | | -54 | 14 | 26 | left inferior frontal (pars opercularis) |
|  |  | | 6.15 | | -46 | 22 | 24 | left inferior frontal (pars triangularis) |
|  |  | | 6 | | -40 | 20 | -4 | left inferior frontal (pars orbitalis) |
|  |  | | 4.75 | | -50 | 14 | -10 | left temporal pole |
|  |  | |  | |  |  |  |  |
| 0 | 1575 | | 6.43 | | -2 | -32 | 32 | left posterior cingulate |
|  |  | | 6.05 | | -10 | -68 | 34 | left precuneus |
|  |  | | 4.91 | | -6 | -22 | 22 | left cuneus |
|  |  | | 4.7 | | -8 | -14 | 32 | left middle cingulum |
|  |  | | 3.4 | | -10 | -44 | 4 | left calcarine |
|  |  | |  | |  |  |  |  |
| 0 | 557 | | 5.88 | | -28 | -68 | 46 | left inferior parietal |
|  |  | |  | |  |  |  |  |
| 0.008 | 148 | | 5.38 | | -66 | -30 | 6 | left middle temporal |
|  |  | | 3.28 | | -62 | -14 | 8 | left superior temporal |
|  |  | |  | |  |  |  |  |
| 0.019 | 125 | | 4.61 | | 62 | -20 | 2 | right superior temporal |
|  |  | |  | |  |  |  |  |
| 0.002 | 195 | | 4.2 | | 38 | 22 | -6 | right insula |
|  |  | | 3.91 | | 36 | 28 | 6 | right insula |
|  |  | |  | |  |  |  |  |
| 0.041 | 104 | | 3.87 | | -6 | -84 | 2 | left calcarine |
|  |  | |  | |  |  |  |  |
| **Dutch > Japanese** | | |  | |  |  |  |  |
| 0 | 2482 | | 6.97 | | 58 | -44 | 40 | right supramarginal |
|  |  | | 5.14 | | 56 | -50 | 26 | right angular |
|  |  | | 4.56 | | 48 | -50 | 10 | right middle temporal |
|  |  | |  | |  |  |  |  |
| 0 | 1886 | | 6.82 | | -48 | -64 | 16 | left middle temporal |
|  |  | | 5.9 | | -58 | -44 | 44 | left inferior parietal |
|  |  | | 4.89 | | -56 | -44 | 34 | left supramarginal |
|  |  | | 4.79 | | -24 | -86 | 36 | left superior occipital |
|  |  | | 4.38 | | -54 | -56 | 26 | left angular |
|  |  | | 3.94 | | -36 | -82 | 36 | left middle occipital |
|  |  | |  | |  |  |  |  |
| 0 | 525 | | 4.84 | | 6 | -36 | 50 | right middle cingulum |
|  |  | | 4.12 | | 8 | -54 | 46 | left precuneus |
|  |  | |  | |  |  |  |  |
| 0.002 | 191 | | 4.53 | | 6 | 64 | 10 | right superior medial frontal |
|  |  | |  | |  |  |  |  |
| 0.006 | 160 | | 4.15 | | 42 | 4 | 4 | right insula |
|  | |  | 4.02 | | 50 | 0 | 8 | right rolandic operculum |
|  | |  | 3.23 | | 34 | 8 | -2 | right putamen |

# Supplementary Table 4. Group differences

| Cluster |  | Peak |  |  |  |  |
| --- | --- | --- | --- | --- | --- | --- |
| p-value | size | z-value | X | y | z | AAL location |
| **Young > Teen** | |  |  |  |  |  |
|  |  |  |  |  |  |  |
| 0 | 297 | 5.04 | 56 | -46 | 48 | right inferior parietal |
|  |  | 3.54 | 62 | -46 | 26 | right supramarginal |
|  |  |  |  |  |  |  |
| 0.003 | 167 | 4.03 | 34 | 30 | 44 | right middle frontal |
|  |  |  |  |  |  |  |
| 0.023 | 111 | 3.85 | 30 | 44 | 32 | right middle frontal |
|  |  |  |  |  |  |  |
| **Teen > Young** | |  |  |  |  |  |
|  |  |  |  |  |  |  |
| 0.003 | 165 | 4.77 | -2 | 16 | 50 | left supplementary motor area |
|  |  |  |  |  |  |  |
| 0 | 295 | 4.61 | -40 | 10 | 26 | left inferior frontal (pars triangularis) |
|  |  |  |  |  |  |  |
| 0.048 | 93 | 3.61 | -32 | -76 | 46 | left inferior parietal |

# Encoding and training of Japanese words

## Encoding

Training began with the encoding of the 30 to-be-learned Japanese words. Each word was presented auditorily, while a picture depicting the word and a definition of the word in Dutch were presented on the screen (Figure1.B). Shortly after the presentation of the word, the definition was presented through the speaker. Each item (word, picture, and definition) was presented once. Pictures were either from list 1 or 2, and the choice of the list was counter-balanced across participants.

## Cued-recall task (training)

After encoding, six blocks of a cued-recall task followed to enhance word-meaning associations. In this task, children heard the encoded Japanese words through the speaker, one at a time, and had to choose a corresponding picture/definition. In the odd-numbered blocks, four pictures from the encoded list were presented on the screen, one of which corresponded to the word. Children were instructed to choose the corresponding picture by pressing the corresponding key associated with the correct picture. In the even-numbered blocks, three short definitions of the trained Japanese words were presented, and the task was to choose the correct definition by pressing the corresponding key. After each trial, the word was repeated auditorily and the correct response option (picture/definition) was displayed on the screen. At the end of each block the number of correct responses in that block was shown to the children.

## Word repetition task (training)

A word repetition task followed the cued-recall task. Children heard a Japanese word through the speaker and saw the corresponding picture on the screen. The children were instructed to repeat the word aloud. No feedback was given. Their voice responses were recorded but not analyzed.

## Picture naming task (training)

Next was the naming task, in which children were instructed to name the Japanese object shown on the screen. All 30 pictures from the training were presented one at a time, and children had 10 s to respond. They were instructed to press the space button after each overt response, after which the correct word was presented auditorily. If the child did not know the answer, they had the option to press the button to hear the correct answer without responding, and if the child did not respond within 10 s, the correct response was presented. Their voice responses were recorded but not analyzed.

## Free recall (training)

To test their word-form memory, children were asked in the free recall test to recall as many trained Japanese words as possible and speak them out within a time limit of three minutes. Responses were recorded and scored offline. For each response, three points were given if the word was pronounced perfectly, two points if the word sounded very similar to the trained word (one or two phoneme difference), and one point was given to any word that sounded similar to one of the trained words. The sum of the weighted score ((number of trials coded as correct x 3) + (number of trials coded as very similar x 2) + (number of trials coded as similar x 1)) was used as the performance measure.

# Behavioral results – additional

Given that individual differences such as vocabulary size and working memory span are known to affect learning and consolidation of novel words, we could expect consolidation/integration trajectories to differ between individuals. Child participants were tested on their working memory span (forward and backward digit span, word list repetition), vocabulary size (Peabody Picture Naming Task: PPVT, Dutch version), and logical thinking (Raven’s Progressive Matrices) on Day0 as a part of screening procedure. Because word span working memory score correlated highly with PPVT (*r=* .295, *p=* .042) and digit span working memory (*r=* .345, *p=* .016), we included normalized PPVT score, digit span (normalized score of forward and backward digit span as tested in Wechsler Intelligence Scale for Children), and percentile score of Raven’s Progressive Matrices as covariates for the behavioral tests (free recall, association test, lexical decision and semantic priming).

## Free recall

A repeated measures ANOVA with within-factor *Time* (Day1, Day8) and between-factor *Group* (Young, Teen) was tested. PPVT, digit span and Raven’s scores were included as covariates of interest. This revealed a main effect of Group (Teen better than Young, *F(1,41)* = 21.06, *p* < .001, *η_p_^2^* = .339), and a trend for an interaction effect Time by PPVT score (*F(1,41)* = 3.16, *p* = .083, *η_p_^2^* = .072). Children with high vocabulary size were likely to remember more words at delayed test. Digit span score also correlated with free recall performance in general (*F(1,41)* = 5.19, *p* = .028, *η_p_^2^* = .112) showing a better performance for the children with higher digit span scores.

## Association memory

A repeated measures ANOVA with within-factors *Time* (Day1, Day8) and *Condition* (Same. Similar), and between-factor *Group* (Young, Teen) was tested. PPVT, digit span and Raven’s scores were included as covariates of interest.

### Accuracy

Similar to the analysis without covariates, a 2-way interaction Condition × Group (*F(1,43)* = 7.27, *p* = .010, *η_p_^2^* = .15) as well as a main effect of Group (*F(1,43)* = 51.37, *p* < .001, *η_p_^2^* = .54) was observed. A main effect of time was no longer observed (*p=* .264) and a main effect of Condition showed only a trend (*F(1,43)* = 3.66, *p* = .062, *η_p_^2^* = .08). Furthermore, accuracy score interacted with PPVT score (*F(1,43)* = 4.48, *p* = .040, *η_p_^2^* = .094) and also showed a trend with Raven’s score (*F(1,43)* = 3.17, *p* = .082, *η_p_^2^* = .07). Overall, the Teens were better than the Young group, and Young group performed worse on the Similar condition relative to the Same condition when compared to the Teens. The children with high intelligence scores (PPVT, Raven) did better than those with lower scores.

### Reaction time

When covariates were added, the main effects Time were no longer observed (*p= .*963). A main effect of Group was still significant (Teens better than Youngs; *F(1,41)* = 8.62, *p* = .005, *η_p_^2^* = .167). A main effect of Condition (Same better than Similar) was observed as a trend (*F(1,41)* = 3.37, *p* = .073, *η_p_^2^* = .073). None of the covariates affected the reaction time score (*p >* .284).

## Lexical decision (in the scanner)

A repeated measures ANOVA with factors Time (Day1, Day8), Language (Japanese, Dutch) and Group (Young, Teen) with covariates PPVT, digit span and Raven’s scores were tested.

### Accuracy

A main effect of Group (Teens better than Youngs; *F(1,43)* = 34.35, *p* < .001, *η_p_^2^* = .444) and 3-way interaction Time by Language by Group showed a significant effect (*F(1,43)* = 10.24, *p* = .003, *η_p_^2^* = .192). Furthermore an interaction effect Time by digit span score was significant (*F(1,43)* = 9.12, *p* = .004, *η_p_^2^* = .175). Overall accuracy performance increase from Day1 to Day8 was observed more for the children with lower digit span scores.

### Reaction time

A main effect of Group (Teens faster than Youngs; *F(1,43)* = 11.57, *p* = .001, *η_p_^2^* = .212) and a 2-way interaction Language by Group showed a significant effect (*F(1,43)* = 4.50, *p* = .040, *η_p_^2^* = .095). A 3-way interaction Time by Language by Group showed a trend (*F(1,43)* = 3.41, *p* = .072, *η_p_^2^* = .073). Furthermore an interaction effect Time by digit span score was significant (*F(1,43)* = 6.99, *p* = .011, *η_p_^2^* = .140). Reaction time decreased more for the children with lower digit span scores.

## Semantic priming

Only RTs of correct responses were included in the analysis, and RTs above or below 2 SDs of the participant’s mean were excluded from the analysis (5 % for Young, 4 % for Teen). Mean RTs of each condition were analyzed by a repeated measures ANOVA with factors Time (Day1, Day8), and Group (Young, Teen). Half of the target words appeared for the first time in the related condition, and the other half of the target words appeared for the first time in the unrelated condition. Due to possible repetition effects, we further included Order (related condition as first, unrelated condition as first) as a factor in the model. Furthermore, covariates PPVT, digit span and Raven’s scores were added to the model as well.

The analysis revealed a trend for a 2-way interaction Time by Group (*F(1,43)* = 3.97, *p* = .053, *η_p_^2^* = .085) and a significant interaction effect Time by digit span score (*F(1,43)* = 4.55, *p* = .039, *η_p_^2^* = .096). Increase in priming effect (i.e. decrease in negative priming effect) was greater for the children with lower digit span scores.
